# Analysis of N^7^-(2-carbamoyl-2-hydroxyethyl)guanine in dried blood spot after food exposure by Ultra High Performance Liquid Chromatography–Tandem Mass Spectrometry

**DOI:** 10.1186/s13065-022-00875-1

**Published:** 2022-11-02

**Authors:** Yahdiana Harahap, Winning Bekti Safitri, Sunarsih Sunarsih

**Affiliations:** 1 Faculty of Pharmacy, Universitas Indonesia, Depok, Indonesia; 2 Faculty of Military Pharmacy, Republic of Indonesia Defense University, Sentul, Bogor Indonesia; 3 Dea Medica Clinic, Bogor, Indonesia

**Keywords:** N^7^-(2-carbamoyl-2-hydroxyethyl)guanine, Acrylamide, Dried blood spot, UHPLC-MS/MS

## Abstract

N^7^-(2-carbamoyl-2-hydroxyethyl)guanine (N7-CAG) is a DNA adduct formed by glycidamide, which is the metabolite of acrylamide. Acrylamide can be found in foods containing reducing sugars and asparagine that are heated at high temperatures. Analysis of N7-CAG was performed in Dried Blood Spot (DBS) samples from 25 subjects of group test who consumed a lot of acrylamide-containing foods and 25 subjects of negative control group. This study aimed to determine whether there is a significant difference in the levels of N7-CAG between the two groups. DBS samples were extracted using the QIAamp DNA Mini Blood Kit and analyzed using Ultra High Performance Liquid Chromatography–Tandem Mass Spectrometry (UHPLC-MS/MS). Separation was performed using an Acquity UPLC BEH C_18_ column (2.1 mm × 100 mm; 1.7 μm), eluted a flow rate of 0.1 ml/min under an isocratic of mobile phase of 0.1% formic acid and acetonitrile. The bioanalytical method of N7-CAG in DBS with allopurinol as the internal standard by using UHPLC-MS/MS has been validated. The calibration curve range of N7-CAG obtained was 10–300 ng/ml with a coefficient of correlation of 0.997. The results of the analysis on 25 test group subjects showed that the concentration of N7-CAG ranged from 1.87 to 23.71 ng/ml, while the 25 subjects in the negative group ranged from 1.18 to 8.47 ng/ml. The results of the Mann Whitney test showed that there was a significant difference in the levels of N7-CAG between the test group and the negative control group with p value less than 0.001.

## Introduction

N7-CAG is a major DNA adduct product due to exposure to acrylamide. Acrylamide is a substance commonly used in its polymer form in the water industry, paper industry, textile industry, and reagents for laboratory purposes [1]. Since 2002, acrylamide has been found in several food products such as biscuits, cereals, bread, French fries, chips, popcorn, and coffee in high quantities [2]. According to the International Agency for Research on Cancer (IARC), acrylamide belongs to group 2A carcinogens, which are probably carcinogenic to humans [3]. Acrylamide can be formed naturally in foods containing reducing sugars and the amino acid asparagine through the Maillard reaction at high temperatures. [4]. Acrylamide that enters the body can be converted to glycidamide by CYP2E1 in phase I metabolism. Glycidamide can interact with DNA to form DNA adducts which can cause mutations and cause inhibition during transcription and DNA replication, leading to cell death or abnormal cells [5]. Therefore, analysis of N7-CAG was performed to predict how much DNA modification results from exposure to acrylamide-containing foods.

Research on the analysis of acrylamide has been done previously on DBS student subjects who often consume foods containing acrylamide using UHPLC-MS/MS. The study proved that there was a significant difference in blood acrylamide levels between students who consumed foods containing acrylamide and negative controls [6]. To determine how much DNA modification is caused by exposure to acrylamide from food, it is necessary to conduct research on the analysis of N7-CAG which is the result of lesions formed in DNA.

Analysis of N7-CAG with allopurinol as an internal standard in DBS using UHPLC-MS/MS has been developed previously and has been fully validated. This research is the application of a bioanalytical method to determine the amount of N7-CAG in humans due to exposure to acrylamide from food.

## Materials and methods

### Chemical and materials

N7-CAG purchased from Santa Cruz Biotechnology (United States of America), allopurinol as an internal standard is obtained from Jiangsu Yew Pharm (China), Dried Blood Spot Card Whatman 903 purchased from Sigma Aldrich (United States of America), QIAamp DNA Blood mini kit was purchased from QIAGEN (Germany), acetonitrile and formic acid were purchased from Merck (Germany), ultrapure water was processed using the Arium^®^ pro Ultrapure Water System from Sartorius (United States of America), and whole blood was purchased from Palang Merah Indonesia (Jakarta, Indonesia).

### Preparation of stock solutions, calibration samples and quality control samples

The stock solution of N7-CAG and allopurinol as an internal standards were prepared by dissolving these compounds in ultrapure water so that each concentration was 1000 µg/ml. The standard solution for the calibration curve was prepared by diluting the stock solution of N7-CAG to a concentration range of 10–300 ng/ml. The internal standard solution was prepared in a concentration of 1 µg/ml. Standard solutions for quality control were prepared by diluting the stock solution of N7-CAG to concentrations of 10 ng/ml (LLOQ), 30 ng/ml (QCL), 150 ng/ml (QCM), and 225 ng/ml (QCH).

### Sample preparation

Blood containing 50 µL of the sample was pipetted and spotted on DBS Whatman 903 paper, then allowed to dry for 3 h at room temperature. DBS was cut according to the width of the spots and put into the sample cup. Samples were extracted using the QIAamp DNA Blood Mini Kit. The extraction procedure refers to the protocol in the QIAamp DNA Mini and Blood Mini Handbook [7]. The extract was hydrolyzed with a mixture of ultrapure water and 90% formic acid in the same ratio (50 µL) and heated for 60 min at 90 °C. After the solution temperature was equal to room temperature, 10 µL of the solution was injected into the UHPLC-MS/MS system.

### UHPLC-MS/MS equipment and conditions

This research was performed by Ultra High Performance Liquid Chromatography—Tandem Mass Spectrometry (Waters, Xevo Triple Quadrupole) consisting of the Quaternary Solvent Manager Acquity^®^ UPLC H-Class (Waters, USA); FTN Acquity^®^ UPLC Sample Manager (Waters, USA); nitrogen gas generator (PEAK Scientific); Acquity^®^ UPLC BEH C18 (1.7 m, 100 mm × 2.1 mm) column (Waters, USA); mass analyzer in the form of triple quadrupole Xevo TQD with Zspray TM ionization source (Waters, USA); and data processing software (MassLynx Software, USA).

The analytical conditions in the UHPLC-MS/MS system applied in this study used a mixed mobile phase of acetonitrile solution and 0.1% formic acid solution with isocratic elution. The flow rate of the mobile phase was adjusted to 0.1 ml/min and the column temperature was 50 °C. The injection volume was 10 µL with a total run time of 4 min. The temperature of the desolvation gas was set at 349 °C and the rate of the desolvation gas used was 645 L/h. The voltage at the inlet was 32 V with the gas rate at the inlet was 10 L/h and the capillary tube voltage was 2.98 kV. The voltage in the collision chamber for N7-CAG was 18 V and for allopurinol was 20 V. The ionization method used in ESI mode was positive (+) and the sample was detected by multiple reaction monitoring (MRM). The MRM transition used for the analysis of N7-CAG was m/z 238.97 > 152.06 and m/z 136.9 > 110 for the allopurinol.

### Partial validation of analytical method

The method has been validated based on the Bioanalytical Method Validation guidelines from the US Food and Drug Administration in 2018. In this study, partial validation was carried out consisting of calibration curve, linearity, accuracy, and precision. The linearity and range parameters are carried out by analyzing at least 6 concentration levels, blank samples which are sample matrix without the addition of analytes and internal standard, and zero sample which is a sample matrix that is only added by internal standard. The concentration range of the calibration curve for N7-CAG, is 10–300 ng/ml. The data obtained from the analysis using the UHPLC-MS/MS system are the analyte peak area and the internal standard. The data is used to make a calibration curve so that the linear regression equation and its correlation coefficient can be obtained. The accuracy (%diff) of each level concentration must be calculated. Intra-day accuracy and precision parameter tests were performed by analyzing at least 4 levels of sample concentration with 5 replicas each in a single run and then the value of %diff and coefficient variation (%CV) were calculated to determine the accuracy and precision.

### Application of the method

This research was reviewed and approved by Health Research Ethics Committee, Faculty of Medicine, Universitas Indonesia, Jakarta, Indonesia, (KET-40/UN2F1.ETIK/PPM.00.02/2021). In this study, there were two groups of subjects, namely the test group and the negative control group, each group totaling 25 subjects. The inclusion criteria of the test subjects were aged 18–56 years, consumed a lot of foods containing acrylamide such as potato chips, popcorn, bread, coffee, cereals, and biscuits, were not active or passive smokers, and signed the informed consent form after being given an explanation as a sign of willingness to participate in the study. Inclusion criteria for negative control subjects were healthy people, aged 15–56 years, rarely or did not eat foods containing acrylamide such as potato chips, popcorn, bread, coffee, cereals, and biscuits, willing to not consume foods containing acrylamide within 96 h before sampling, and signed an informed consent form after being given an explanation as a sign of willingness to participate in the study. The subject’s blood was taken through the peripheral vein at the fingertip using a lancet as much as 100 µL. Then, 50 µL blood sample was spotted on DBS Whatman 903 paper using a calibrated micropipette and allowed to be absorbed into the paper to dry for 3 h at room temperature. After drying, the DBS paper was stored in a plastic clip containing silica gel and placed in an ice box [8].

### Statistical analysis

Statistical analysis was carried out on the results of the sample analysis to determine whether there were significant differences between the two groups. The Mann Whitney test was chosen as a statistical test in this study because the data from the analysis were not normally distributed.

## Results and discussion

### Chromatography and sample preparation

Sample preparation is carried out on DBS Whatmann 903 using the QIAamp DNA Blood Mini Kit with the aim of isolating the DNA contained in blood cells. Then, hydrolysis is carried out to free the adducts from nucleotides or nucleosides so that they can be analyzed [9]. UHPLC-MS/MS is chosen as the analytical instrument in this study, because it has high sensitivity to detect very small compounds and also has high selectivity so that it can analyze compounds in complex matrices such as blood [10, 11]. This study used UPLC BEH C18 Acquity^®^ column to separate N7-CAG and allopurinol from the interference of biological matrices with a total analysis time of 4 min. The retention time for N7-CAG was 2.75 min and allopurinol 3.07 min.

### Partial validation

The analytical method used in this study has been previously validated. In this analytical method there are no modifications. Moreover, the laboratories, instruments, and tools used are also the same. Therefore, in this study only partial validation was carried out to ensure that this method still meets the requirements based on the FDA and EMA guidelines even though it is carried out by different analysts.

### Calibration curve and linearity

**Fig. 1:**
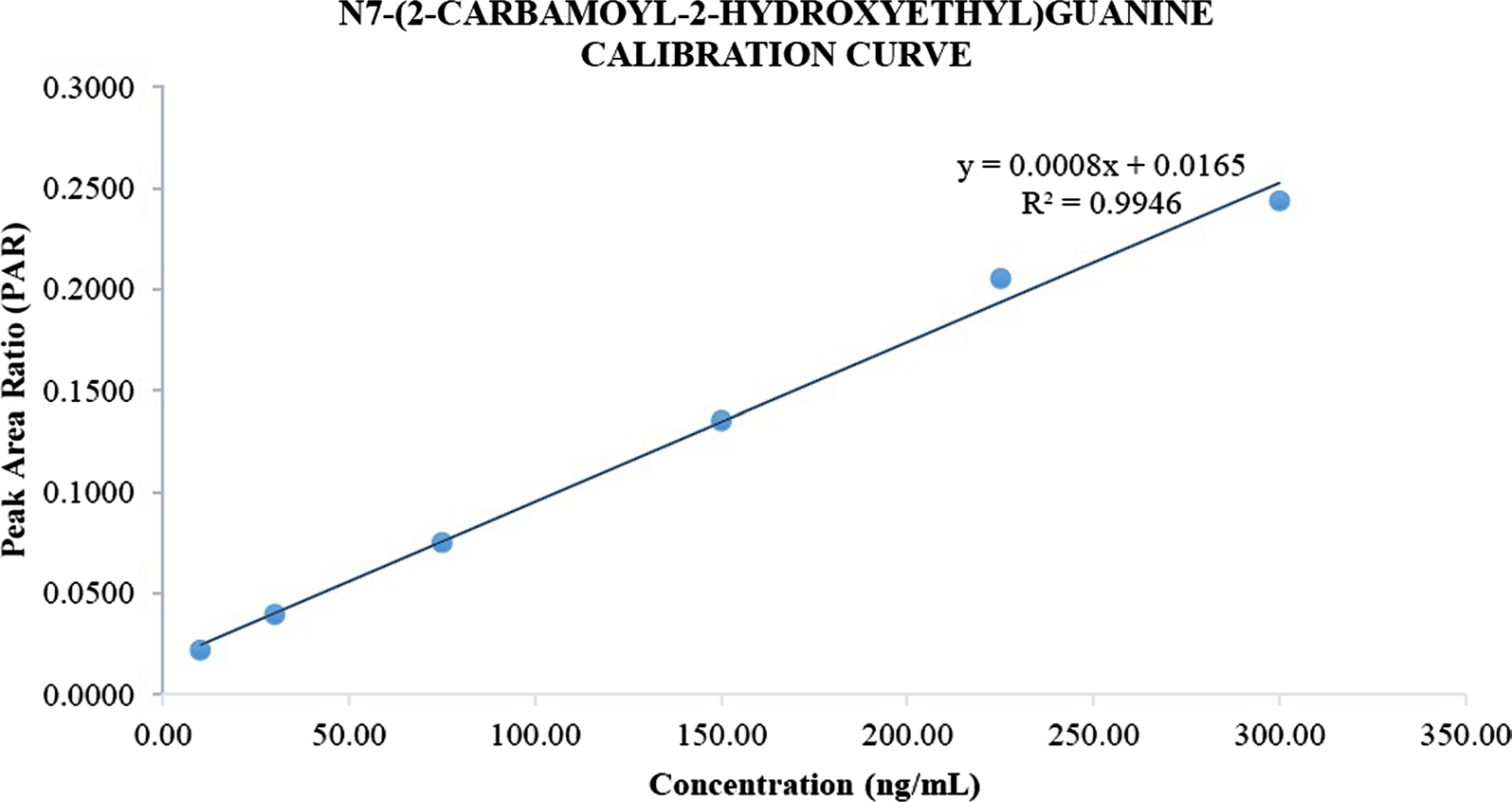
N7-CAG Calibration Curve

The relationship between analyte concentration and analyte response after injection should be tested. Each concentration level is added to the matrix and then sample preparation is carried out for further analysis with UHPLC-MS/MS. In this study, the concentration range of the calibration curve of N7-CAG, which is 10–300 ng/ml with six levels of concentration, zero sample, and blank sample. The calibration curve for N7-CAG can be shown in Fig. 1. The correlation coefficient (r) that can be acceptable for the calibration curve made in the biological matrix should be more than 0.990 [12]. In this study, the correlation coefficient obtained exceeds the acceptability value of 0.990. The calibration curve obtained is linear and meets the requirements.

In addition, the recalculation of the concentration from the calibration curve is carried out. Each concentration level must be within the range of ± 15% of the nominal value, except for LLOQ it must be within the range of ± 20% of the nominal value [13]. In this study, the %diff value obtained on the calibration curve of N7-CAG is 9.64% for LLOQ and 4.23 to 9.82% for other concentrations. The %diff value obtained meets the requirements and the calibration curve of N7-CAG can be used in this bioanalysis method.

### Within-run accuracy and precision

Accuracy is a parameter that describes how close the measured analyte concentration is to the actual analyte concentration and is expressed in %diff value. While precision is a parameter that can indicate how close the repeatability of the results obtained from the bioanalysis method is and expressed in the value of %CV. Within-run accuracy and precision tests are carried out using the concentration of LLOQ, QCL, QCM, and QCH with 5 replicas each in a single run. In this study, within-run accuracy for N7-CAG has met FDA requirements with a %diff value not more than ± 15% at the concentration levels of QCL, QCM, and QCH and LLOQ not more than ± 20% of the actual concentration. In addition, the within-run precision for N7-CAG has also met FDA requirements with a %CV value is no more than 15%, except for an LLOQ concentration is no more than 20%. Whithin-run accuracy and precision of N7-CAG are shown in Table 1.

**Table 1:** Whithin-run accuracy and precision of N7-CAG

Sample	Actual concentration (ng/ml)	Measured concentration (Average ± SD; ng/ml)	CV (%)	%diff
LLOQ	10.00	10.05 ± 0.41	4.07	− 5.10 to 4.59
QCL	20.00	26.67 ± 1.29	4.84	− 13.96 to − 3.49
QCM	150.00	138.37 ± 3.35	2.42	− 10.35 to − 4.90
QCH	225.00	213.90 ± 11.53	5.39	− 12.26 to 2.08

### Analysis of N7-CAG in dried blood spot after food exposure

Before collecting the sample, this research has received a certificate of passing the ethical clearance from the Health Research Ethics Committee, Faculty of Medicine, Universitas Indonesia with the number KET-40/UN2F1.ETIK/PPM.00.02/2021. In this study, there were two groups of subjects, namely the test group and the negative control group, each of which consisted of 25 people. The subjects of the test group and the negative control group must meet the inclusion and exclusion criteria of the study and fill out a questionnaire before sampling is carried out.

**Fig. 2:**
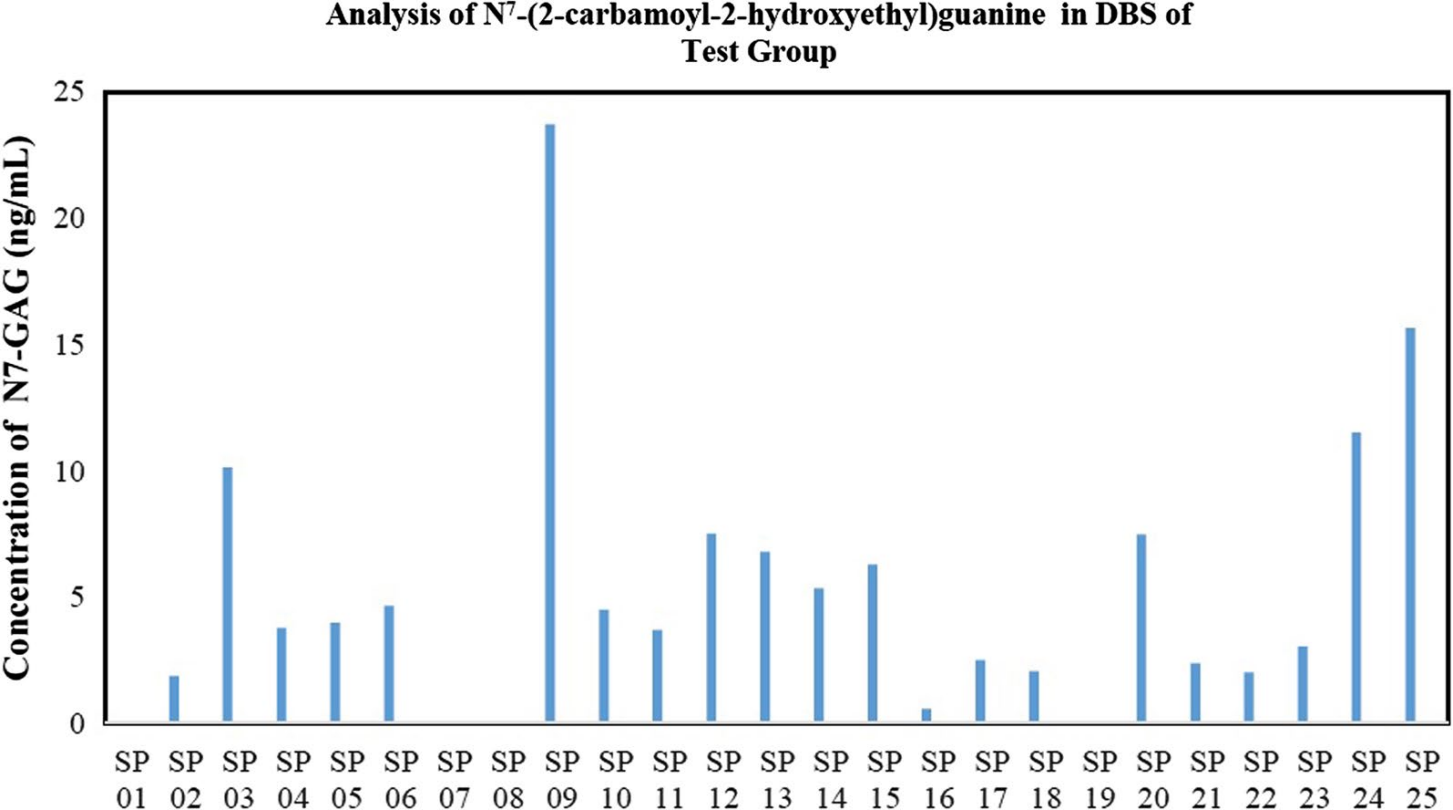
Graph analysis of N7-CAG in DBS of 25 test group

Based on the results of the analysis on 50 subjects, 46 subjects have levels of N7-CAG are below the LLOQ value. The LOD value obtained through statistical calculations is 0.65 ng/ml [14]. The results of the analysis on 25 test group subjects showed that there is N7-CAG in 4 subjects that could be detected quantitatively. The Graph analysis of N7-CAG in DBS of the test group can be shown in Fig. 2. The highest levels are obtained in subjects with code SP 09, which is 23.71 ng/ml. Meanwhile, the lowest level that can be quantified is in the subject with the code SP 03, which is 10.13 ng/ml. The lowest level of N7-CAG that could still be detected in the DBS samples of the test subjects is 1.87 ng/ml. The habit of consuming food and beverages containing acrylamide with a concentration of N7-CAG in the test subject group is shown in Table 2.

**Table 2:** The habit of consuming food and beverages containing acrylamide with a concentration of N7-CAG in the test subject group

Subject Code	Habit of consuming food and drink									Measured concentration of N7-CAG (ng/ml)
	Chips	French Fries	Coffee	Fried food	Fast food	Pop Corn	Cereal	Biscuit	Bread	
SP 01	2	2	3	3	3	3	3	3	3	0*
SP 02	3	2	2	1	2	1	0	0	1	1.87
SP 03	1	2	3	1	1	1	1	1	1	10.13
SP 04	3	1	4	1	1	1	1	2	2	3.77
SP 05	3	3	1	2	2	3	1	3	3	3.98
SP 06	1	2	0	1	2	1	2	2	4	4.65
SP 07	2	2	2	1	2	1	2	2	2	0*
SP 08	2	3	2	3	2	1	1	2	2	0*
SP 09	4	4	4	2	1	0	0	4	1	23.71
SP 10	3	2	0	2	1	0	1	2	2	4.49
SP 11	4	4	1	2	2	2	0	4	2	3.70
SP 12	4	4	1	2	2	2	0	4	2	7.51
SP 13	2	1	0	1	1	1	0	1	1	6.79
SP 14	2	1	1	1	1	1	0	2	1	5.34
SP 15	2	1	3	1	2	1	3	2	2	6.28
SP 16	4	1	4	1	1	0	1	4	1	0.58*
SP 17	3	3	1	3	4	1	0	4	4	2.49
SP 18	4	3	3	2	3	3	0	4	4	2.06
SP 19	2	2	2	1	1	1	1	1	1	0*
SP 20	2	2	0	2	2	1	1	2	2	7.47
SP 21	2	1	2	2	2	1	1	2	2	2.37
SP 22	2	1	1	2	1	1	2	2	2	2.02
SP 23	3	3	1	3	3	3	3	3	3	3.04
SP 24	3	3	2	2	3	2	1	2	3	11.50
SP 25	3	2	4	2	3	2	1	2	3	15.64

Description: Chips include potato chips, cassava, tempeh, maicih, bananas, peanut brittle and onions

Fried foods include combro, misro, molen, pastel, risoles, tempe mendoan or tempe flour, flour fried tofu, fried cassava, bakwan, crispy mushrooms, and cireng

Fast food includes burgers, pizza, hot dogs, nuggets, and fried chicken/shrimp/fish

Bread includes white bread, dry bread, toast, and dry bread

4 = Very often

3 = Often

2 = Sometimes

1 = Rarely

0 = Never

*Below LOD which is 0.65 ng/mL

**Fig. 3:**
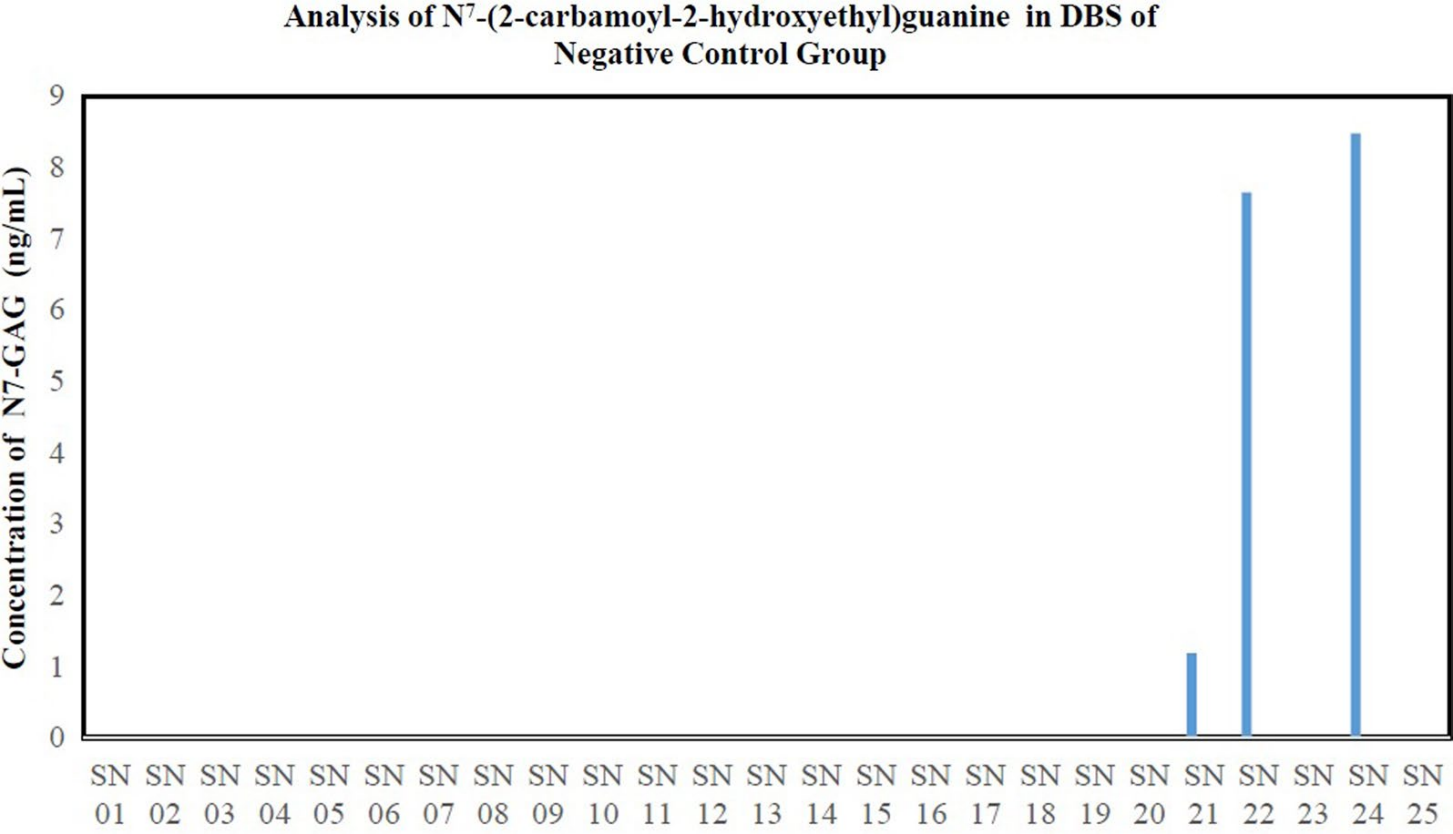
Graph analysis of N7-CAG in DBS of 25 negative control group

The results of the analysis on 25 subjects in the negative control group show that all samples are below the LLOQ value and 3 subjects are detectable. The Graph analysis of N7-CAG in DBS of the negative control group can be shown in Fig. 3. The level of N7-CAG that can be detected is 1.18 ng/ml. The habit of consuming food and beverages containing acrylamide with a concentration of N7-CAG in the negative control group can be seen in Table 3. Negative control group subjects are enrolled in this study as a comparison to the test group so that it can be obtained the data on the significant difference in levels of N7-CAG between the test group and the negative control group. In the Mann Whitney test, the p-value obtained is less than 0.001 so that the conclusion obtained is that there is a significant difference in the levels of N7-CAG between the test group and the negative control group. The data from the Mann Whitney test is shown in Table 4.

**Table 3:** The habit of consuming food and beverages containing acrylamide with a concentration of N7-CAG in the group of negative control subjects

Subject Code	Habit of consuming food and drink									Measured concentration of N7-CAG (ng/ml)
	Chips	French Fries	Coffee	Fried food	Fast food	Pop Corn	Cereal	Biscuit	Bread	
SN 01	2	1	2	1	1	0	0	1	1	0*
SN 02	2	1	0	1	2	1	0	2	2	0*
SN 03	0	1	4	1	1	0	0	1	2	0*
SN 04	1	1	0	1	1	0	0	1	1	0*
SN 05	1	1	0	1	2	0	0	0	2	0*
SN 06	1	1	0	1	1	1	1	1	1	0*
SN 07	2	2	0	1	1	0	0	2	2	0*
SN 08	1	1	1	1	1	1	1	1	1	0*
SN 09	1	1	1	1	2	1	1	1	2	0*
SN 10	2	1	1	1	1	1	1	1	2	0*
SN 11	1	1	0	1	1	1	1	1	2	0*
SN 12	1	1	0	1	2	0	0	1	1	0*
SN 13	1	1	0	1	2	0	1	1	1	0*
SN 14	1	1	0	1	1	0	0	1	1	0*
SN 15	1	1	0	1	1	0	0	0	1	0*
SN 16	1	1	1	1	2	0	0	1	1	0*
SN 17	1	1	1	1	1	1	0	1	1	0*
SN 18	1	1	0	1	2	0	0	1	1	0*
SN 19	1	1	0	1	1	1	1	1	1	0*
SN 20	1	1	0	1	1	0	0	1	1	0*
SN 21	1	1	0	2	1	0	0	1	1	1.18
SN 22	1	1	0	1	1	0	0	1	1	7.64
SN 23	1	1	0	1	0	1	1	1	1	0*
SN 24	1	1	0	1	2	0	0	1	1	8.47
SN 25	1	1	0	1	1	0	0	1	1	0*

Description: Chips include potato chips, cassava, tempeh, maicih, bananas, peanut brittle and onions

Fried foods include combro, misro, molen, pastel, risoles, tempe mendoan or tempe flour, flour fried tofu, fried cassava, bakwan, crispy mushrooms, and cireng

Fast food includes burgers, pizza, hot dogs, nuggets, and fried chicken/shrimp/fish

Bread includes white bread, dry bread, toast, and dry bread

4 = Very often

3 = Often

2 = Sometimes

1 = Rarely

0 = Never

*Below LOD which is 0.65 ng/mL

**Table 4:** Mann Whitney test results data

Group	Median (minimum–maximum)	*P* value
Test (*n* = 25)	3.77 (0.00–23.71)	< 0.001*
Negative Control (*n* = 25)	0.00 (0.00–8.47)	

*Significant difference

Another study on the analysis of N7-CAG was conducted by Huang et al., in 2014 [15]. The sample of the biological matrix used in the study was urine collected from smokers and nonsmokers. Then the analysis was carried out using UHPLC-MS/MS at its optimum conditions to obtain data on the levels of N7-CAG. The average level of N7-CAG in urine in smoking subjects was 2.01 ng/ml. Meanwhile, in non-smoker subjects, the average level of these compounds was 1.5 ng/ml urine. In this recent study, the sample of the biological matrice used for the analysis of N7-CAG is blood which obtained from a peripheral vein of the subject. The results of the analysis showed that the levels of N7-CAG in the blood is higher when compared to urine. The lowest concentration that can be detected in the DBS samples is 1.18 ng/ml, while the highest concentration that can be quantified in the DBS samples is 23.71 ng/ml. One of the largest component in the blood is red blood cells in which there is DNA in large number. Glycidamide which is electrophilic can interact with DNA in the blood so that it can form N7-CAG. Some DNA adducts take time to separate from the DNA strand to form apurination sites [16]. This study showed that the compound N7-CAG is still found bound to DNA strands in the blood.

## Conclusion

This study showed that there is a significant difference in the levels of N7-CAG between the test group and the negative control group. The results of this study indicate that DBS can be used as the biosampling technique to determine N7-CAG levels due to exposure to acrylamide from food or drink.

## Data Availability

The clinical data used to support the findings of this study are included within the article.
